# Defining and Improving Outcomes Measurement for Virtual Care: Report from the VHA State-of-the-Art Conference on Virtual Care

**DOI:** 10.1007/s11606-023-08464-1

**Published:** 2024-01-22

**Authors:** Samantha L. Connolly, Scott E. Sherman, Navid Dardashti, Elizabeth Duran, Hayden B. Bosworth, Michael E. Charness, Terry J. Newton, Ashok Reddy, Edwin S. Wong, Leah L. Zullig, Jeydith Gutierrez

**Affiliations:** 1 Center for Healthcare Organization and Implementation Research (CHOIR), VA Boston Healthcare System, Boston, MA USA; 2 Department of Psychiatry, Harvard Medical School, Boston, MA USA; 3 Virtual Care Consortium of Research (VC CORE), VA New York Harbor Healthcare System, New York, NY USA; 4 Department of Population Health, NYU Grossman School of Medicine, New York, NY USA; 5 Center of Innovation to Accelerate Discovery and Practice Transformation (ADAPT) Durham Veterans Affairs Medical Center, Durham, NC USA; 6 Department of Population Health Sciences, Duke University Medical Center, Durham, NC USA; 7 Chief of Staff of the VA Boston Healthcare System, Boston, MA USA; 8 Department of Neurology, Harvard Medical School, Boston, MA USA; 9 Department of Neurology, Boston University Chobanian & Avedisian School of Medicine, Boston, MA USA; 10 Director of Clinical Analytics, VA Office of Connected Care, Washington, DC, USA; 11 General Medicine Service, VA Puget Sound Health Care System, Seattle, WA USA; 12 Department of Medicine, University of Washington School of Medicine, Seattle, WA USA; 13 Center of Innovation for Veteran-Centered and Value-Driven Care, VA Puget Sound Health Care System, Seattle, WA USA; 14 Department of Health Systems and Population Health, University of Washington, Seattle, USA; 15 Center for Access and Delivery Research, Iowa City VA Healthcare System, Iowa City, IA USA; 16 Department of Internal Medicine, University of Iowa, Iowa City, IA USA

## Abstract

Virtual care, including synchronous and asynchronous telehealth, remote patient monitoring, and the collection and interpretation of patient-generated health data (PGHD), has the potential to transform healthcare delivery and increase access to care. The Veterans Health Administration (VHA) Office of Health Services Research and Development (HSR&D) convened a State-of-the-Art (SOTA) Conference on Virtual Care to identify future virtual care research priorities. Participants were divided into three workgroups focused on virtual care access, engagement, and outcomes. In this article, we report the findings of the Outcomes Workgroup. The group identified virtual care outcome areas with sufficient evidence, areas in need of additional research, and areas that are particularly well-suited to be studied within VHA. Following a rigorous process of literature review and consensus, the group focused on four questions: (1) What outcomes of virtual care should we be measuring and how should we measure them?; (2) how do we choose the “right” care modality for the “right” patient?; (3) what are potential consequences of virtual care on patient safety?; and (4) how can PGHD be used to benefit provider decision-making and patient self-management?. The current article outlines key conclusions that emerged following discussion of these questions, including recommendations for future research.

## INTRODUCTION

Virtual care, which includes synchronous and asynchronous telehealth, remote patient monitoring, and the collection and interpretation of patient-generated health data (PGHD), was available long before the onset of the COVID-19 pandemic.^1, 2^ However, prior to COVID-19, virtual care was primarily utilized within specific populations, such as those living in rural areas with limited access to local specialty care.^3^ Virtual care use dramatically increased during the pandemic, spurred by regulatory and reimbursement changes that were rapidly adopted to allow more care to be provided from a distance.^4–6^ While this shift towards virtual care offers potential benefits, such as greater access to care for patients, much remains to be learned regarding its impact on outcomes of care, health equity, and the future of healthcare delivery more broadly.^2^

The rise in virtual care has led to a growing body of research reporting positive outcomes, including high patient satisfaction^7, 8^, reduced travel costs^9^, and the successful management of chronic conditions from a distance.^10^ However, there is still much to be learned regarding the specific clinical scenarios in which virtual care is most effective. Virtual care outcomes have been inconsistently defined and reported in the literature, making it challenging to assess the generalizability of specific studies or interventions. As such, it is imperative that virtual care outcomes are appropriately measured and reported across populations, including patients with multiple chronic conditions and complex social needs. As we plan for care provision beyond the pandemic, there is a need for increased research regarding the safe delivery of virtual care, as well as how to effectively integrate remote visits into broader care processes. Collectively, this work will help to ensure that all patients are receiving high-quality care and will serve to inform future virtual care policies and best practices.

The Veteran’s Health Administration (VHA) is the largest integrated healthcare system in the USA and is a leader in virtual care, making it an ideal setting in which to examine these critical questions regarding outcomes of care. The VHA Office of Health Services Research and Development (HSR&D) convened a State-of-the-Art (SOTA) Conference on Virtual Care to develop research priorities and an agenda to advance virtual care provision. The SOTA Conference was organized by the VHA HSR&D Virtual Care Consortium of Research, a grant-funded program which aims to strengthen collaborations between VHA virtual care researchers. Participants were divided into workgroups to address three main focus areas: access; engagement; and outcomes of virtual care. In this article, we outline the process undertaken by the Outcomes Workgroup and the resulting consensus recommendations for research priorities that were developed.

## METHODS

In preparation for the SOTA conference, an Outcomes Planning Committee was formed, including one clinician researcher lead (SES) and two administrative staff members (ND and ED) who were located at one of the main study sites of the Virtual Care Consortium of Research, as well as two additional clinician researchers (SLC and JG) who were active members of the consortium and were identified as subject matter experts in virtual care outcomes research. The Outcomes Planning Committee met regularly for 9 months prior to the SOTA conference, during which they were periodically joined by a Veteran representative, selected for her experiences receiving VHA virtual care. Through a series of teleconferences, they worked together to develop a list of additional experts to invite to the SOTA conference Outcomes Workgroup who represented a breadth of experience and perspectives regarding virtual care. Nine of these experts ultimately participated, including one clinician researcher, three health services researchers, the national director of VHA HSR&D, three operational partners from the VHA Office of Connected Care, and one VHA healthcare system chief of staff; the Veteran representative was unable to attend. The five Outcomes Planning Committee members also participated, leading to a total of 14 workgroup members. All workgroup members were VHA employees, and all authors of the current paper were workgroup members.

The Outcomes Planning Committee agreed on using the National Academy of Medicine domains of high-quality care as a guiding framework. The National Academy of Medicine defines high-quality care as being safe, timely, effective, efficient, equitable, and patient-centered.^11^ The committee iteratively refined the key questions to be addressed by the SOTA participants and selected pre-conference readings. The committee then developed an evidence brief, informed by the National Academy of Medicine quality domains, summarizing the most relevant literature on virtual care outcomes. This brief was based on a rapid review of the literature, which did not aim to be exhaustive but compiled current evidence across a range of fields and applications of virtual care. The evidence brief ultimately contained information from three original research articles and 16 reviews.

In addition, the Outcomes Planning Committee developed a set of three goals that the workgroup would be tasked to address. The full-day, in-person Outcomes Workgroup meeting during the SOTA conference had the following goals:

1. Identify virtual care outcome areas with sufficient evidence (i.e., areas which include multiple moderate or high-quality studies reporting similar conclusions across key outcome variables), such that further research would not be a priority;
2. Identify virtual care outcome areas that would benefit from additional research to guide clinical practice and policy; and
3. Identify high-impact research areas for VHA, including those more likely to impact the clinical care of Veterans and those in which VHA is uniquely positioned to evaluate the outcomes of interest as the largest integrated healthcare system in the USA.

The evidence brief and goals were distributed to all Outcomes Workgroup participants in advance of the conference to inform the discussion. During the first day of the SOTA conference, all participants attended an initial briefing by key operational and research partners regarding the current state of virtual care at VHA and the conference objectives. Following this meeting, each of the workgroups met separately to deliberate and reach consensus, when possible, on research priorities for their specific topics (access, engagement and outcomes). The Outcomes Workgroup underwent a brainstorming process to propose questions worth exploring; the participants were instructed to generate questions independently, after which each member shared their ideas aloud and engaged in group discussion. This process led to the creation and refinement of 11 unique questions after accounting for overlapping themes. Workgroup members then voted and selected the four highest ranked questions to be discussed in subgroups. The remainder of the day was spent in two breakout sessions, each of which was divided into two subgroups, allowing two questions to be discussed in each session. At the end of each breakout session, the two subgroups reconvened and delivered brief presentations of their findings to the larger group, followed by a discussion period. The Outcomes Planning Committee members served as facilitators and recorders of workgroup discussions. On the second day of the SOTA, each workgroup presented their findings to the rest of the SOTA attendees. Participants then had the opportunity to discuss and vote on research priority recommendations for VHA.

Following completion of the SOTA conference, the members of the Outcomes Planning Committee reviewed all notes recorded from the Outcomes Workgroup and drafted the current manuscript, which reports on the key conclusions from the group. After an initial manuscript draft was developed, it was circulated to all members of the Outcomes Workgroup for review and editing. The final draft of the manuscript was approved by all workgroup members.

## RESULTS

The workgroup chose the following four questions to examine: (1) What outcomes of virtual care should we be measuring and how should we measure them?; (2) how do we choose the “right” care modality for the “right” patient?; (3) what are potential consequences of virtual care on patient safety?; and (4) how can patient-generated health data (PGHD) be used to benefit provider decision-making and patient self-management? Key conclusions that emerged following discussion of these four questions are outlined below.

### Question 1: What Outcomes of Virtual Care Should We Be Measuring and How Should We Measure Them?

The group discussed existing outcome measures for virtual care as well as the strength of the evidence supporting their use in specific circumstances. There was consensus regarding the abundance of evidence supporting the use of virtual care for the management of specific chronic conditions and diseases like diabetes, hypertension, heart failure, and depression, among others.^12, 13^ Patient and provider satisfaction with virtual care has also been extensively documented.^7, 8^ Most studies have been designed as non-inferiority studies in specific patient cohorts, diseases, or conditions and often report on only one outcome (e.g., satisfaction, cost, or a clinical outcome). This limits the applicability of these findings to real-world patient populations and a larger healthcare system like VHA, in which roughly one-third of patients have three or more chronic conditions.^14^ Furthermore, although many studies have reported clinically significant patient outcomes, variability in outcome measurement makes it difficult to compare findings across modalities, interventions, and conditions.^15^

**Figure 1:**
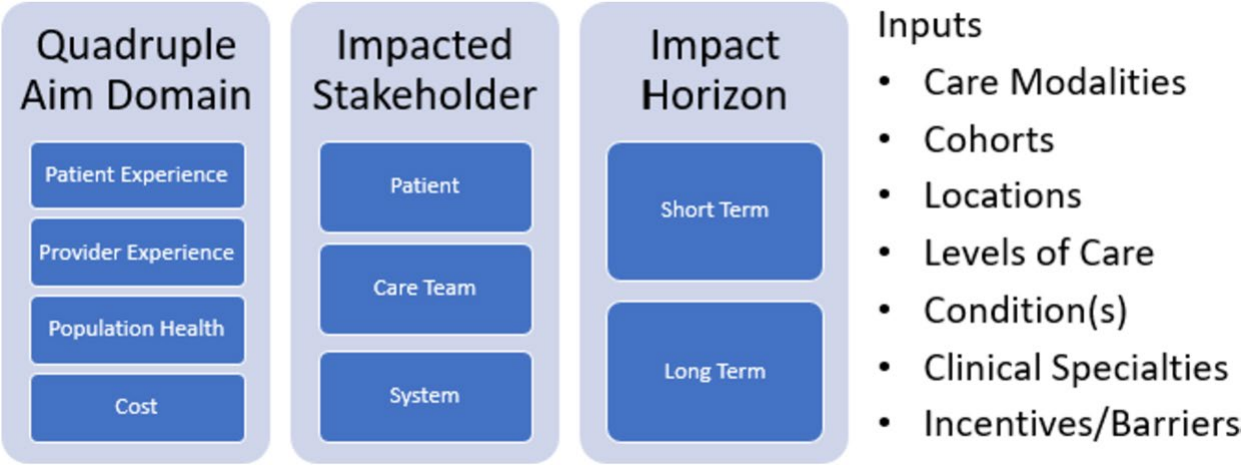
Proposed framework for evaluating virtual care outcomes.

The group’s recommendations focused on ways to improve the definition of care quality and virtual care outcome measures. A holistic, systematic approach and standard set of measures would allow researchers to assess the true value of virtual care and advance evidence-based recommendations. The group proposed a framework for evaluating virtual care outcomes at three levels: (1) the quadruple aim domains of the patient experience of care, provider experience, population health, and cost^16^; (2) the impacted populations, including patients, care teams, and healthcare systems; and (3) the impact horizon or timeframe of the outcome (Fig. 1). This standard framework would allow for different inputs (e.g., care modality, level of care, clinical condition) to be compared across the continuum of care in a standardized and systematic manner.

The group concluded that it is important to use consistent measures across studies to allow for comparison of outcomes and identification of trends and patterns. Attention must be paid to appropriate measurement, and new outcome measures should only be developed if validated instruments or measures do not already exist. Feedback should also be solicited from the patient and clinical communities regarding which outcomes are most relevant to them and if certain outcomes require the development of new measures.

### Question 2: How Do We Choose the “Right” Care Modality for the “Right” Patient?

The group agreed that there is a need for additional research to identify the specific scenarios in which virtual care can be leveraged to improve patient outcomes. The effectiveness and appropriateness of virtual care will vary as a function of patient characteristics, including diagnosis, access to care, and comfort with technology.^17, 18^ At present, much of our understanding of virtual care outcomes is derived from either restrictive randomized controlled trials or observational studies in which patients may be excluded due to having multiple comorbidities or social and environmental challenges.^12^ While these studies were fundamental in developing an initial evidence base for the field of virtual care, there is a need to evaluate how these care modalities can be utilized by real-world patient populations to improve health outcomes. As such, future research should test the outcomes of virtual care among complex patient populations, such as high healthcare utilizers and those with multiple chronic or high-risk conditions. Patients with challenging social and environmental factors, such as minimal social support, low health literacy, low income, and/or limited internet access, should also be included. It is important to prioritize these patients to gain a more ecologically valid understanding of the effectiveness of virtual care.

Additionally, it will be important to examine hybrid models of care to help determine the ideal ratios of in-person versus virtual clinical interactions based on patient needs. This work must include a clear focus on processes of care and how these processes may differ based on care modality. Potential areas of inquiry could include whether patients are less likely to have their vital signs measured or to receive follow-up lab work if they are seen virtually as opposed to in-person, and the impact, if any, of such variation on health outcomes. Some work has begun in this space, including findings that patients with diabetes were less likely to have their A1C measured when they were being treated virtually, but that this lack of measurement did not have a short-term impact on A1C levels or other diabetes-related outcomes.^10^ Another study found that patients who had completed virtual visits had comparable or improved performance on most included quality measures (e.g., influenza vaccination, lipid panels) as compared to patients treated entirely in-person.^19^

Additional research regarding the use of hybrid models will be critical in determining whether virtual visits should function primarily as periodic check-ins before patients can receive certain examinations or procedures in-person, or whether virtual care can fully replace in-person care in specific clinical scenarios. It will be particularly important to track patients who have been treated virtually over longer follow-up periods to see if potential differences in outcomes emerge at greater time intervals. Collectively, this research will help providers to make more informed decisions regarding the best ways to treat their patients, balancing patient preference for virtual care with rigorous data demonstrating its clinical effectiveness relative to in-person visits.

### Question 3: What Are Potential Consequences of Virtual Care on Patient Safety?

There is little published research examining the potential impacts of virtual care on patient safety. Large healthcare systems such as VHA are well suited to conduct these types of studies, given that they have larger patient populations in which to detect relatively low-incidence adverse events. It will be important to identify whether virtual care introduces novel safety concerns that are unique to remote care, such as potential breaches of videoconferencing platform security, or whether adverse outcomes are similar to those encountered during in-person care. To this end, the group discussed six domains of safety impacted by virtual care delivery within the framework developed by the Agency for Healthcare Research and Quality (AHRQ): referrals, care transitions, testing, diagnostic errors, prescribing and medication errors, and safety culture in health systems.^20, 21^ Studies of referrals and care transitions will be critical to help understand whether hand-offs and consultation requests are less likely to be successful when completed virtually as opposed to on-site. Future research should examine whether there is a greater risk of communication failures when visits are conducted remotely as compared to in-person. For instance, less tech-savvy patients may be unable to access test results or post-visit instructions via a patient portal following a telehealth visit but would have no difficulty when handed a paper report in-person; soliciting patient preferences and mailing follow-up documentation to those who prefer hard copies may help to improve outcomes of virtual visits for certain populations.

Future work should also assess whether the quality of the clinical encounter may be compromised in some virtual visits, which aligns with the AHRQ domains of testing, diagnostic errors, and prescribing and medication errors. Specifically, there is a need for more work examining whether there are differences in the receipt of guideline-concordant testing when patients are seen virtually versus in-person. In addition, patients who do not own a video-enabled device may be treated via an audio-only phone visit, which may lead to lower quality care and potentially an incorrect diagnosis as the provider is unable to visually assess the patient’s health status.^22–24^ Future studies should examine potential differences in diagnostic and prescribing accuracy between virtual and in-person encounters and whether this varies by condition; some work has begun in this area and has reported mixed results.^25^

When seeking to study virtual care safety outcomes, one must first identify the potential safety concern in question. It will be important to conduct qualitative research with providers and patients to understand the potential safety risks associated with virtual care. Given the stigma around acknowledging adverse events, it is likely that many errors that have occurred in the context of virtual care have not been widely reported; indeed, a large proportion of adverse events occurring during studies of healthcare interventions are never published.^26^ Thus, careful attention to the AHRQ domain referencing the safety culture within a given healthcare system will be critical. Once a safety concern has been identified, it must be measured; for instance, the frequency with which inaccurate diagnoses are made during virtual versus in-person assessments. The use of pre-existing measures may be appropriate or in some cases, a new virtual care-specific safety measure may need to be developed. If virtual care is found to pose safety risks, it may then be appropriate to create an intervention to address the concern. This could include developing provider guidance or regulations regarding what types of care can be provided virtually versus in-person. Collectively, this work will be critical in ensuring that virtual care is used appropriately and only in the circumstances where it is clinically effective and safe.

### Question 4: How Can PGHD Be Used to Benefit Provider Decision-making and Patient Self-management?

There is growing interest in the use of PGHD to inform clinical care and engage and empower patients in managing their health. The group divided PGHD into three categories: unsolicited, suggested, and solicited. Table 1 contains working definitions and examples for each. Solicited PGHD are generally used within formalized remote patient monitoring programs, whereas suggested PGHD are shared based on a more informal understanding between patients and their providers.^27, 28^ Patients may also voluntarily share unsolicited PGHD from a variety of Bluetooth-enabled devices, such as fitness trackers and smart watches.^29^ Uses of unsolicited PGHD are less defined but have considerable potential; thousands of Veterans currently share their PGHD with VHA, and this cohort is projected to grow rapidly over the coming years.^30^

**Table 1:** Types, Definitions, and Examples of PGHD

Type of PGHD	Working definition	Example
Solicited	PGHD that is shared with a provider by the patient or caregiver for diagnostic purposes or for specific disease monitoring programs. Specific verbal or written consent is obtained to participate	A patient with congestive heart failure weighs themselves at home using a Bluetooth-enabled scale for 30 days after hospital discharge. This solicited PGHD is monitored by a case manager who can reach out to the patient if concerning weight gain is identified
Suggested	Suggested PGHD is collected via a more informal agreement between the patient and provider. A shared decision-making conversation should occur to determine how the data will be incorporated into the patient’s care	A provider is managing a patient with hypertension whose in-office blood pressure (BP) readings have been borderline high. The provider suggests that the patient record their BPs at home using either a manual, automated, or Bluetooth-enabled BP cuff. The patient can share these data at the next visit on paper, digitally between visits by manual entry into a patient portal, or automatically by connecting their device
Unsolicited	PGHD that is independently submitted by a patient or caregiver via a patient portal or in a third-party vendor location without a request from their provider. A shared decision-making conversation should occur between the provider and patient to determine how the data will be incorporated into the patient’s care	A patient connects their wearable biometric device or manually enters PGHD (e.g., blood glucose, weight, BP, pulse oximetry) into a patient portal or app. The patient may wish to discuss these data with their provider, or alternatively they may want to monitor their data independently as part of their own health management

Patients may be able to view their health data via app dashboards as well as through patient portals. As this data-sharing capability is relatively new, there is little published research examining how these data may be presented to patients to increase self-management of their health, as well as to providers to create clinically significant value.^31–33^ Similarly, there is little existing research on whether PGHD, combined with traditional data sources such as electronic health records, can be analyzed via artificial intelligence or machine learning technologies to generate predictive insights, alerts, or decision support for clinicians and health systems. Such integration presents a substantial opportunity to target and customize care delivery to patients and in turn improve outcomes. However, additional work is also needed to better understand the accuracy of patient-derived data (e.g., home blood pressure cuff readings), as this will have major impacts on its potential utility.^34^

As one of the largest gatherers of PGHD, VHA has great potential to study whether the sharing of PGHD can have long-term health impacts as well as what patient populations and conditions may benefit most from these technologies.^30, 35^ VHA has multiple ongoing initiatives to integrate PGHD into patient care. For example, Veterans can transfer EKG data from an Apple device to a provider through VHA’s My HealtheVet patient portal, and they can text blood pressure readings to their care team via the VHA’s Annie app.^30^ As VHA and other academic research organizations embark on future PGHD research, the following considerations will be critical: (1) the acceptability to both patients and providers of clinical recommendations based on PGHD; (2) the validity of data generated by rapidly evolving devices; and (3) the protection of the unprecedented amount of protected health information gathered in the process. All of these factors will have major influences on PGHD’s ability to impact outcomes at the patient, provider, and system levels.

## CONCLUSIONS

The Outcomes Workgroup of the VHA State-of-the-Art Conference on Virtual Care underwent a rigorous process of literature review and consensus-building to arrive at four key questions for in-depth discussion: (1) What outcomes of virtual care should we be measuring and how should we measure them?; (2) how do we choose the “right” care modality for the “right” patient?; (3) what are potential consequences of virtual care on patient safety?; and (4) how can PGHD be used to benefit provider decision-making and patient self-management.

The group agreed that future work must move away from observational studies and controlled trials of highly restrictive samples that are not representative of real-world patient populations. More comprehensive approaches that measure multiple levels of virtual care outcomes and that include patients with comorbid conditions and complex social needs will be critical. Researchers should not conceptualize virtual care as a one-size-fits-all modality, but should instead begin to ask more nuanced questions regarding the specific clinical scenarios and patient populations that may benefit most from the integration of virtual care into treatment. Future work should seek to identify optimal proportions of virtual and in-person care based on evidence and patient preference.

Careful consideration of the safety of virtual care is also much needed. Large healthcare systems such as VHA are uniquely positioned to conduct evaluations regarding virtual care safety and can identify potentially low-incidence safety events associated with virtual visits. It will be important to identify whether virtual care introduces new safety concerns unique to the specific technologies being utilized, or whether risks are similar to those encountered during in-person visits. Embedding rigorous measurement and monitoring of safety risks into the design and evaluation of virtual care interventions will serve to improve the quality of virtual care delivery. Finally, the group explored the great promise of PGHD to improve health outcomes and equip both patients and providers with actionable health information that can inform treatment. As the largest integrated healthcare system in the USA, VHA is an ideal setting in which to study the optimal incorporation of PGHD into care.

Collectively, the Outcomes Workgroup of the Virtual Care SOTA conference identified areas in which there is sufficient evidence for virtual care outcomes, areas that would benefit from additional research, and areas that are particularly well-suited to be examined within VHA. The existing outcomes literature demonstrates the potential for virtual care to dramatically transform healthcare delivery. Future research must strive to be nuanced, cross-cutting, and representative of real-world patient populations to ensure the provision of high-quality virtual care for the long term.
